# Cytokine Receptors—Regulators of Antimycobacterial Immune Response

**DOI:** 10.3390/ijms23031112

**Published:** 2022-01-20

**Authors:** Magdalena Druszczyńska, Magdalena Godkowicz, Jakub Kulesza, Sebastian Wawrocki, Marek Fol

**Affiliations:** 1 Department of Immunology and Infectious Biology, Institute of Microbiology, Biotechnology and Immunology, Faculty of Biology and Environmental Protection, University of Lodz, Banacha 12/16, 90-237 Lodz, Poland; magdalena.godkowicz@edu.uni.lodz.pl (M.G.); sebastian.wawrocki@gmail.com (S.W.); marek.fol@biol.uni.lodz.pl (M.F.); 2 Lodz Institutes of the Polish Academy of Sciences, The Bio-Med-Chem Doctoral School, University of Lodz, 90-237 Lodz, Poland; 3 Department of Internal Diseases and Clinical Pharmacology, Medical University of Lodz, Kniaziewicza 1/5, 91-347 Lodz, Poland; jakub.kulesza@icloud.com; 4 Swiss Institute of Allergy and Asthma Research (SIAF), University of Zurich, 7265 Davos, Switzerland

**Keywords:** cytokine, cytokine receptors, immune response, mycobacteria

## Abstract

Cytokine receptors are critical regulators of the antimycobacterial immune response, playing a key role in initiating and coordinating the recruitment and activation of immune cells during infection. They recognize and bind specific cytokines and are involved in inducing intracellular signal transduction pathways that regulate a diverse range of biological functions, including proliferation, differentiation, metabolism and cell growth. Due to mutations in cytokine receptor genes, defective signaling may contribute to increased susceptibility to mycobacteria, allowing the pathogens to avoid killing and immune surveillance. This paper provides an overview of cytokine receptors important for the innate and adaptive immune responses against mycobacteria and discusses the implications of receptor gene defects for the course of mycobacterial infection.

## 1. Introduction

*Mycobacterium tuberculosis* (*Mtb*), a causative agent of tuberculosis (TB) is distinguished by a number of unique characteristic features. The pathogen can live and proliferate extracellularly both in vivo and in vitro, its cell envelope is mostly composed of a complex of lipids and carbohydrates, its extraordinary ability to persist within host macrophages leads to the dormancy of tubercle bacilli [1,2]. The interplay between the pathogen and the innate and adaptive immune cells is multifaceted and includes cytokine/chemokine-mediated host defenses [3]. *Mtb* penetrates the pulmonary alveolus through delivery of droplets containing the bacteria. The size of droplets, to some extent, determines the efficiency of host colonization by *Mtb*. The 0.5–5 micrometer droplets, in contrast to those of 5–10 micrometers, are considered to be more effective carriers of mycobacteria for several reasons: they persist in the air for 2 to 40 h, they are transmitted over a greater distance, they are more efficiently inhaled into the tracheobronchial tree and alveolar space [4]. *Mtb* interacts with airway epithelial cells (AEC), alveolar type II pneumocytes, alveolar macrophages, dendritic cells (DC) and neutrophils. The airway epithelial cells, alveolar type II pneumocytes and alveolar macrophages are the first to be infected by mycobacteria [5]. They respond by producing early mediators of inflammation: tumor necrosis factor (TNF)-α, interleukin (IL)-1α, IL-1β, interferon (IFN)-γ and chemoattractants, among them IL-8 (CXCL8), which recruits neutrophils and monocytes from blood vessels, as well as macrophages and DC [5]. Arriving at the site of infection, DC acquire mycobacterial antigens through the ingestion of *Mtb* or products of its degradation, and by engulfing the apoptotic bodies formed from dying neutrophils and macrophages, containing both living and dead *Mtb* [5,6]. Contact with *Mtb* antigens promotes the production of IL-12 and IFN-β by DC. At the early stage of infection, IFN-β regulates the secretion of IP-10 (CXCL10), a chemokine that attracts NK cells [5]. Via pattern recognition receptors (PRR), such as Toll-like receptors (TLR) and nucleotide-binding oligomerization domain (NOD) proteins, antigen-presenting cells (APCs), including DC and macrophages, identify *Mtb* ligands, for instance, lipoproteins and glycolipids, which results in the production of inflammatory cytokines and chemokines [7]. Infected APCs migrate to local draining lymph nodes where they initiate the development of acquired immunity through antigen presentation to naïve T cells. It has been shown that in mice *Mtb*-specific T cells appear in the lungs 1–3 weeks after infection, which is associated with the IFN-γ production by CD4^+^ T cells and with the control of bacterial burden. Furthermore, IFN-γ producing CD4^+^ T cells provide help for other T cell subsets, including CD8^+^ T cells and γδ T cells, which play an important role in controlling *Mtb* infection [7]. *Mtb* antigens are presented via MHC class II molecules to CD4^+^ T cells leading to their activation. *Mtb* peptides are also presented by MHC class I molecules to CD8^+^ T cells. *Mtb* phosphoantigens are recognized by γδ T cells without APCs support triggering the production of perforin and granzymes, by which they eliminate infected immune cells with *Mtb* inside them. Moreover, they can produce IFN-γ and TNF-α in response to intracellular pathogens [8]. Effector and memory T cells are essential for controlling *Mtb* infection, however, particular attention is focused on the CD4-based immune response, especially on CD4 cells, which secrete IFN-γ since CD8 response seems to play a much more minor role [9]. 

After *Mtb* uptake, DC migrate from lungs to local draining lymph nodes where they present *Mtb* antigens to T cells. T cells migrate back to the site of infection in the lungs and participate in granuloma formation. *Mtb* prevents phagosome-lysosome fusion, which is essential to the survival of this pathogen within mononuclear phagocytes. Additionally, to a certain extent, tubercle bacilli can influence the course of immune response of the host, for example by increasing the production of anti-inflammatory IL-10 [10]. This cytokine can affect the ability of DC and macrophages to activate Th1 cells. Close interaction of macrophages and DC with T lymphocytes CD4^+^ and CD8^+^ is required for the effective control of infection [11,12]. A crucial step in anti-*Mtb* host response is the formation of a granuloma, which represents a pathological hallmark of TB. This structure is an agglomerate of immune cells that walls off the pathogen. It is believed that granuloma may be a manifestation of protective immunity, however, it may also give rise to endobronchial transmission following its necrosis [13,14]. The primary role in granuloma formation is attributed to several effector cytokines, of which TNF-α and IFN-γ are particularly important in TB [7,15]. In the center of granuloma are macrophages, while the peripheral layer is constituted mostly by lymphocytes [6]. The role of TNF is twofold, on the one hand, it limits bacterial growth, on the other hand, its excess in the microenvironment, together with the strong T-cell immunity, may lead to macrophage necrosis and destabilize the granuloma structure resulting in the release of mycobacteria and their uncontrolled proliferation [16]. Newly infected macrophages do not show high antimicrobial activity and only minimally antagonize *Mtb* growth via TNF-dependent mechanisms [16]. By secreting the proinflammatory cytokines IL-1 and IL-6, macrophages and DC play an important role in the recruitment of cells at the site of infection. Newly arriving macrophages contribute to the formation of granulomas while simultaneously expanding the intracellular niche that is permissive for the growth of mycobacteria. Finally, *Mtb*-specific T cells producing IFN-γ, which enhance the microbicidal activity of macrophages, create the granuloma cuff [16]. Another type of immune cells often found in granulomas are neutrophils. It has been found that in vitro, after stimulation with *Mtb* neutrophils produce TNF, IL-4, and IL-10. Similarly, neutrophils presented in a granuloma also express the same cytokine catalog expanded with IFN-γ [17]. Infected neutrophils can produce IL-10, a cytokine that reduces the inflammatory response. It is still debatable whether neutrophils destroy *Mtb* that has been engulfed or if they operate as a “Trojan horse”. However, macrophages that phagocytose dead or dying *Mtb*-infected neutrophils are known to become activated and release TNF-α [5]. What is particularly surprising, the studies conducted in non-human primates showed that T cells producing IFN-γ, TNF, IL-2, IL-17, or IL-10 make up, on average, less than 10% of all lymphocytes in the granuloma. This low percentage of lymphocytes producing canonical T-cell cytokines could explain how granulomas maintain the balance between excessive inflammation and bacterial control [18].

**Figure 1:**
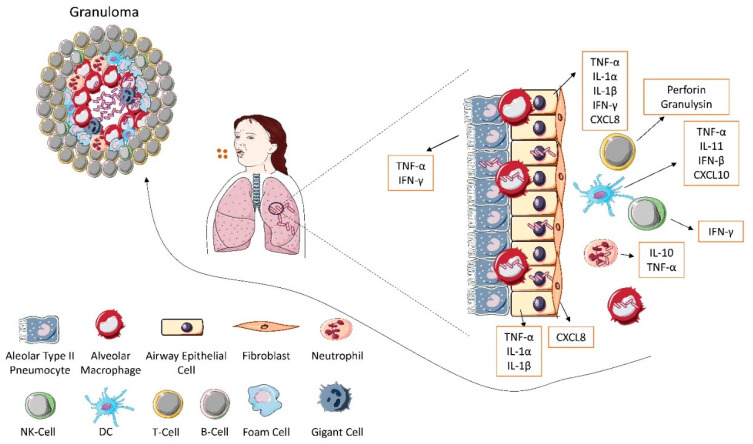
Major cytokines and chemokines involved in antimycobacterial immune response. Cytokines and chemokines provide cell-to-cell communication and play key roles in cell migration and development of immune responses. Cytokine and chemokine production and their ability for modulation of immune cells reactivity through cytokine binding to the specific receptors is essential in the course of immune events during *Mtb* infection. Abbreviations: CXCL8—interleukin 8, CXCL10—interferon-gamma induced 10kDa protein (IP-10), IFN-γ—interferon-gamma, IL—interleukin, TNF-α—tumor necrosis factor-alpha.

Cytokines and chemokines provide cell-to-cell communication and play key roles in cell migration and development of immune responses (Figure 1). Cytokine/chemokine production and their ability for modulation of immune cells reactivity through cytokine binding to the specific receptors is essential in the course of immune events during *Mtb* infection. Herein, we review receptors for cytokines/chemokines that play a primary role in antimycobacterial immune response, including their contribution to disease progression or dormancy. Understanding the function of cytokine/chemokine receptors in response to *Mtb* infection is important in the control of TB.

## 2. Cytokine Receptors Involved in Antimycobacterial Immune Response

Cytokine receptors are membrane-bound or soluble glycoproteins that serve as cytokine docking sites and inductors of a signaling cascade inside the cells. They are involved in the initiation of intracellular signaling that regulates a diverse range of biological functions including metabolism control, neural stem cell activation, inflammatory responses as well as blood cell and immune cell development and growth. The classification of cytokine receptor families is based on the structural homology of the extracellular cytokine binding domains and common intracellular signaling mechanisms. The main families include type I cytokine receptors, type II cytokine receptors, chemokine receptors, the tumor necrosis factor (TNF) receptor family, the transforming growth factor (TGF)-β receptor family, the immunoglobulin (Ig) superfamily, and the interleukin (IL)-17 receptor family (Table 1).

### 2.1. Type I Cytokine Receptors

**Figure 2:**
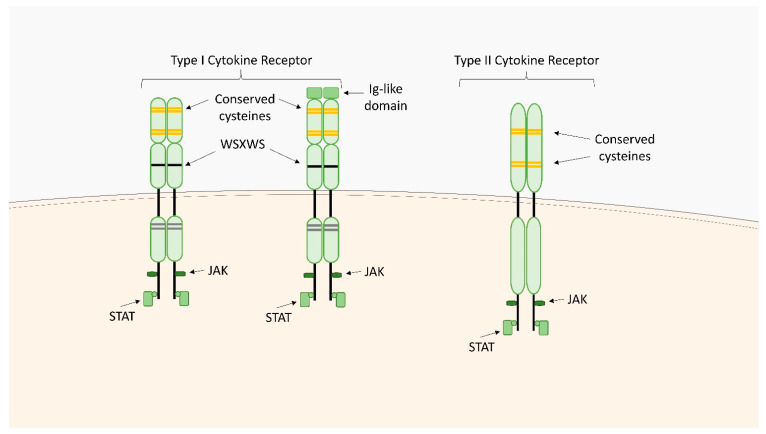
Structure of type I and type II cytokine receptors. The cytokine receptor homology region (CHR) contains four conserved cysteine moieties in the first FnIII domain and a tryptophan-serine-X-tryptophan-serine (WSXWS) motif in the second FnIII domain. Many type I cytokine receptors also contain immunoglobulin (Ig) domains. Cytoplasmic receptor domains contain sites for JAK and STAT binding. Type II cytokine receptors: CHRs have conserved cysteine residues arranged differently and do not contain the WSXWS motif compared to type I cytokine receptors. The cytoplasmic receptor domains contain sites for JAK and STAT binding.

The type I cytokine receptors family includes cell surface expressed transmembrane receptors that recognize four-helix bundle cytokines such as IL-2, IL-4, IL-6, IL-12, IL-23, the granulocyte colony-stimulating factor (G-CSF), and the granulocyte-macrophage colony-stimulating factor (GM-CSF). The unifying feature of these receptors is the lack of intrinsic protein tyrosine kinase activity and transduction of the signaling pathway through the involvement of the non-receptor Janus kinases (JAKs) as well as the signal transducer and activator of transcription (STATs) factors. They are composed of several amino acid chains with conserved intracellular and extracellular features (Figure 2). The extracellular domains contain a region known as the haemopoietin domain or the cytokine receptor homology region (CHR) formed by a pair of Fibronectin type III (FnIII) modules, at the junction of which the primary cytokine binding site is located. CHRs contain four conserved cysteine residues within the first FnIII domain and a tryptophan-serine-X-tryptophan-serine (WSXWS) motif in the second FnIII domain [43]. Cysteines are critical to the maintenance of the structural and functional integrity of the receptors, and the WSXWS sequence serves as a recognition site for protein-protein interactions [44]. Many type I cytokine receptors also contain additional extracellular domains such as immunoglobulin (Ig) domains, extra FnIII domains or even a second CHR [43]. The cytoplasmic receptor domains, containing the Box 1/Box 2 motifs, provide specific docking sites for JAKs and STATs. In between these two motifs, additional binding sites for negative regulators such as the suppressor of cytokine signaling (SOCS) proteins can be found.

Type I cytokine receptors can function as homodimeric or heterodimeric complexes. They usually consist of a ligand-binding chain and one or more signal-transducing chains (common gamma (γ_c_) chain (CD132), common beta (β_c_) chain (CD131), or glycoprotein 130 (gp130)), which may be shared between various receptors within this receptor family [45]. Binding of type I cytokines to their receptors initiates activation of signal transduction pathways including the JAK-STAT pathway as well as additional signaling systems such as the Ras-mitogen-activated protein kinase (MAPK) pathway, and the phosphatidylinositol 3-kinase (PI3K)/protein kinase B (AKT) pathway.

#### 2.1.1. IL-2 Receptor (IL-2R)

**Figure 3:**
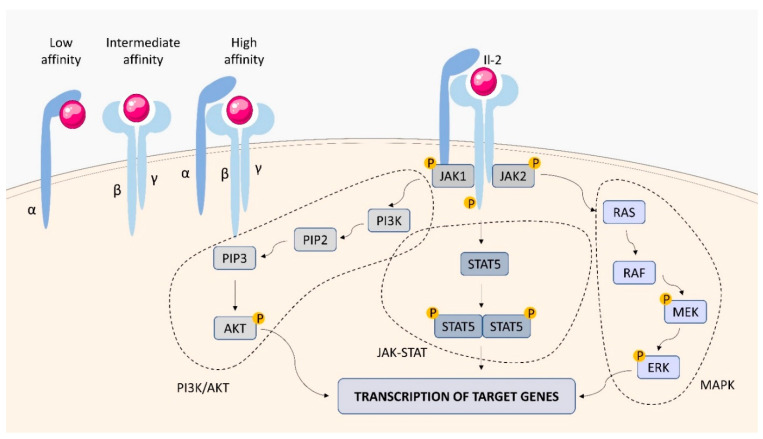
IL-2R structure and signaling. The IL-2 receptor (IL-2R) has three forms, consisting of different combinations of three different chains, IL-2Rα (CD25), IL-2Rβ (CD122), and γc (CD132). The interaction of IL-2 with IL-2R causes phosphorylation of JAK1 and JAK3, which causes downstream signaling through STAT5. The PI3/AKT and MAPK pathways are activated, which stimulates gene transcription in the cell nucleus, leading to cytokine production.

IL-2, known as a major T-lymphocyte growth factor, promotes proliferation and maturation of activated T cells as well as controls B cell proliferation and natural killer (NK) cell cytolytic activity [46]. IL-2 is the first short-chain type I cytokine, for which the receptor structure has been discovered [47]. The functional IL-2 receptor (IL-2R) has three forms, consisting of different combinations of three different chains, IL-2Rα (CD25), IL-2Rβ (CD122), and γ_c_ (CD132) (Figure 3). The IL-2Rβ and γ_c_ subunits belong to the type I cytokine receptor family and are responsible for signal transduction. The chains are expressed separately and differently on various cell types, and can assemble in different combinations, binding IL-2 with low, intermediate, or high-affinity. The IL-2Rα subunit binds IL-2 with low affinity, the combination of the IL-2Rβ and γ_c_ forms an intermediate-affinity IL-2R, and the three-chain complex binds the cytokine with high affinity [47]. IL-2 is first bound by IL2Rα, which leads to a conformational change and increases its affinity for the IL-2Rβ and γ_c_ subunits. IL-2 stimulation induces the activation of the Janus family of tyrosine kinases JAK1 and JAK3, associated with IL-2Rβ and γ_c_, respectively, which phosphorylate IL-2Rβ and induce tyrosine phosphorylation of STATs and various other downstream targets. The downstream signaling pathways activated by IL-2 occur via three major signal transduction systems, the JAK-STAT pathway, the PI3K/AKT pathway, and the MAPK pathway, leading to the transcription of target genes that contribute to IL-2-dependent biological activity [47].

Using the soluble form of the IL-2Rα (sIL-2Rα) as a surrogate marker of IL-2-mediated T cell activation, it was found that HIV infection is associated with low serum levels of sIL-2Rα in patients with TB, even when CD4^+^ lymphocyte counts are relatively well preserved, and that impaired IL-2 signaling could contribute to the profound impact that HIV has had on both the incidence and clinicopathological manifestations of TB [48]. It is suggested that sIL-2R together with IFN-γ and neopterin may serve as parameters to monitor the prognosis of TB, particularly in patients with severe pulmonary TB [49]. IL-2R gene polymorphism analysis including variants of *IL-2RA*, *IL-2RB*, and *IL-2RG* revealed that only *IL-2RA* gene polymorphisms showed a statistically significant association with susceptibility to TB [50]. In the study involving 235 participants with TB and latent Mtb infection, serum levels of IL-2Ra and chemokine CCL1 were higher in TB compared to latently infected individuals; therefore, they can be used as a diagnostic tool to discriminate between these groups [51]. IL-2R appears to have the potential to be used in the therapy of both TB and melanoma by binding fusion toxin composed of the catalytic and transmembrane domains of diphtheria toxin fused to human IL-2, leading to selective depletion of cells expressing the high affinity IL-2 receptor, including regulatory T cells (Tregs). Short-term depletion of IL-2R+ cells has been reported to be beneficial during TB infection, as it results in a decrease in the bacterial burden of the lung and spleen both as monotherapy and as adjunctive therapy administered with standard antibiotic treatment for TB [52].

#### 2.1.2. IL-4 Receptor (IL-4R)

**Figure 4:**
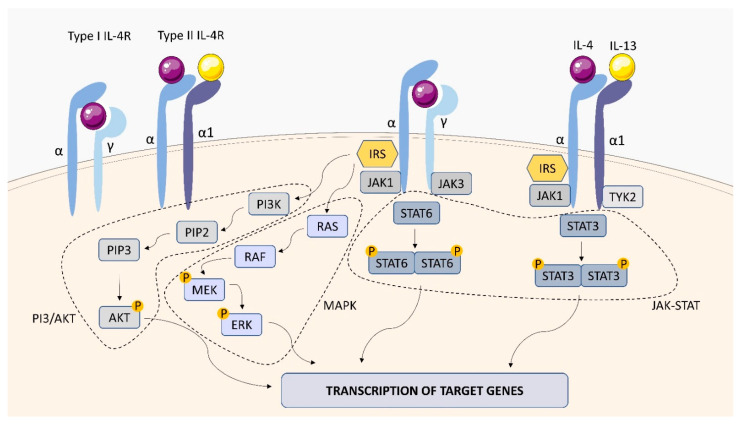
IL-4R structure and signaling. IL-4 receptors consist of three different chains, α, γ and α1. There are two types of IL-4 receptors: type I IL-4R containing α and γ chains, type II IL-4R containing α and α1 (IL-13R) chains. The interaction of IL-4 and IL-13 with IL-4R activates the tyrosine kinases JAK1/JAK3 and TYK2. The PI3/AKT, MAPK and JAK-STAT pathways are activated, which stimulates gene transcription in the cell nucleus, leading to cytokine production.

IL-4 is a pleiotropic type I cytokine that plays a critical role in the regulation of immune response. IL-4 induces the differentiation of naïve CD4 T cells into the type 2 helper (Th2) phenotype, the immunoglobulin (Ig) class switch to IgG1 and IgE in B cells, and alternative macrophage activation [53]. The cytokine exerts its biological activities through interaction with two surface receptor complexes, the type I IL-4R and the type II IL-4R (Figure 4) [54]. Both receptor types contain a common IL-4Rα (CD124) chain, which is a functional receptor subunit. The type I IL-4R is formed by the interaction of the IL-4Rα subunit with the γc subunit (CD132), while the type II IL-4R is formed by the interaction of the IL-4Rα subunit with the IL-13 binding chain, IL-13Rα1 (CD213a1) [55]. The IL-4Rα chain is also a subunit of the IL-13 receptor (IL-13R), which explains the similarity of the biological effects of IL-4 and IL-13. Binding of IL-4 to the IL-4Rα extracellular domain causes a conformational change in the intracellular receptor domains, activating the receptor-associated Janus kinases, which leads to the recruitment of STAT6 and its phosphorylation. Activated STAT6 forms homodimers that translocate to the nucleus and promote transcription of genes responsive to IL-4. Other phosphorylated tyrosine residues bind to proteins with phosphotyrosine binding (PTB) domains including insulin receptor substrate (IRS) proteins. Phosphorylated IRS proteins can subsequently activate the PI3K/AKT signaling pathway or the MAPK cascade [55].

The role of T helper (Th)2 cell-mediated immunity manifested by IL-4 and IL-13 production in the susceptibility and pathogenesis of TB remains a subject of scientific inquiry. Studies carried out in the Ghanaian cohort, in which genotype frequencies of variants of the genes *IL-4*, *IL-13*, *IL-4R*, *IL-13RA1* and *IL-13RA2* were assigned to the size and number of cavities in patients with TB, showed that some variants of IL-4RA and IL-13RA2 are associated with greater risk of cavity development or progression, pointing to a role for both IL-4Rα and the IL-13Rα2 in the pathogenesis and progression of TB [56]. Experiments with the use of mice with IL-13 overexpression (IL-13tg) and with the absence of IL-4Rα (IL-4Rα−/−) revealed that deletion of IL-4Rα abrogates the increased susceptibility of *Mtb*-infected IL-13tg mice and the mandatory role for IL-4Rα in mediating the progression dependent on IL-13 of experimental TB [51]. IL-13 overexpression was found to result in recrudescence of *Mtb* growth accompanied by centrally necrotizing granulomas. It is postulated that the mechanisms driven by IL-13/IL-4Rα are directly related to the development of central granuloma necrosis [57]. Using BALB/c mice deficient in IL-4Rα specifically on B cells (mb1creIL-4Rα−/lox) it was shown that they had decreased mycobacterial burdens and lung pathology during chronic TB infection. It should be stressed that intranasal transfer of IL-4Rα-sufficient B cells isolated from the spleen of wild-type donor mice abolished the protective effect in mb1creIL-4Rα−/lox mice. Furthermore, adoptive transfer of wild-type B cells restored IFN-β production, but not IL-6, IL-12p40, and IL-10 in the lungs of mb1creIL-4Rα−/lox mice. Furthermore, the absence of IL-4Rα on B cells increased the macrophage inflammatory response ex vivo [58].

#### 2.1.3. IL-6 Receptor (IL-6R)

**Figure 5:**
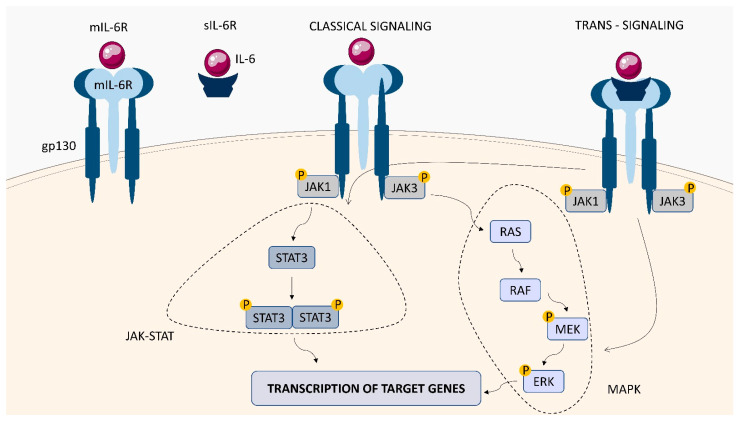
IL-6R structure and signaling. The IL-6-binding receptor complex consists of an IL-6 receptor subunit (IL-6R), existing in both membrane-bound (mIL-6R) and soluble (sIL-6R) forms, and IL-6 signal-transducing chain glycoprotein 130 (gp130). Interaction of IL-6 with IL-6R activates the JAK1/JAK3 tyrosine kinases. The JAK-STAT and MAPK pathways are activated, which stimulates gene transcription in the cell nucleus, leading to cytokine production.

IL-6, originally discovered as a B-cell differentiation factor, is a multifunctional cytokine with extensive immunomodulatory activity. It plays an important role in the regulation of acute phase response, inflammation, immune response, and haemopoiesis [59,60]. The cytokine influences various cell types through its unique receptor system. The IL-6-binding receptor complex consists of an IL-6 receptor subunit (IL-6R), existing in both membrane-bound (mIL-6R) and soluble (sIL-6R) forms, and IL-6 signal transducing chain glycoprotein 130 (gp130) (Figure 5). Although cells that do not express IL-6R do not respond to IL-6 alone, they can be stimulated by the complex formed by IL-6 and sIL-6R [61]. Upon binding of IL-6 to mIL-6R, homodimerization of gp130 is induced to form a high affinity IL-6/IL-6R/gp130 complex, activating a variety of biological signals through two pathways—the JAK/STAT pathway and the MAPK pathway.

Produced primarily by monocyte-derived and recruited macrophages, IL-6 manages (together with IL-1 and TNF-α) the development of the acute phase response in TB. However, *Mtb* can affect the production of this cytokine and, by reducing it, leads to disease progression [62]. Furthermore, *Mtb* can dysregulate IL-6 production through the family of cytoplasmic proteins called suppressors of cytokine signaling (SOCS), leading to excessive IL-6 production in the epithelium, which inhibits STAT signaling [63]. Susceptibility and severity of TB have been reported to be associated with genetic variants in *IL-6/IL-6R* [64]. Studies conducted by Ritter et al. with the use of IL-6- and T-cell-specific gp130-deficient mice showed that the absence of IL-6 or gp130 in T cells has only a minor effect on the development of Th1 and Th17 antigen-specific cells after aerosol infection with *Mtb* [65]. Delgobo et al. provided interesting data that point to the improvement of IL-6R-mediated myeloid differentiation by human CD34+ cells in vitro by live *Mtb* [66]. The use of advanced tools, including ingenuity pathway analysis (IPA), gene set enrichment analysis (GSEA), STRING network analysis of protein-protein interactions, for comprehensive analysis of large transcriptomic and proteomic data sets allowed us to suggest that *Mtb* infection activates a gene module shared by both type I IFN and IL-6, linking downstream “interferon-stimulated genes” (ISG) and lineage-specific regulators of myeloid differentiation—CCAAT/enhancer binding proteins (CEBP). Furthermore, the IL-6/IL-6R/CEBP gene module was found to be a central component correlated with monocyte expansion during *Mtb* infection in vivo, which is amplified in severe pulmonary and systemic disease. The transcriptional induction of CEBPB and CEBPD controlled by IL-6- and type I IFN signaling appears to be a relatively recent event in mammalian and primate evolution [66].

#### 2.1.4. IL-12 Receptor (IL-12R)

**Figure 6:**
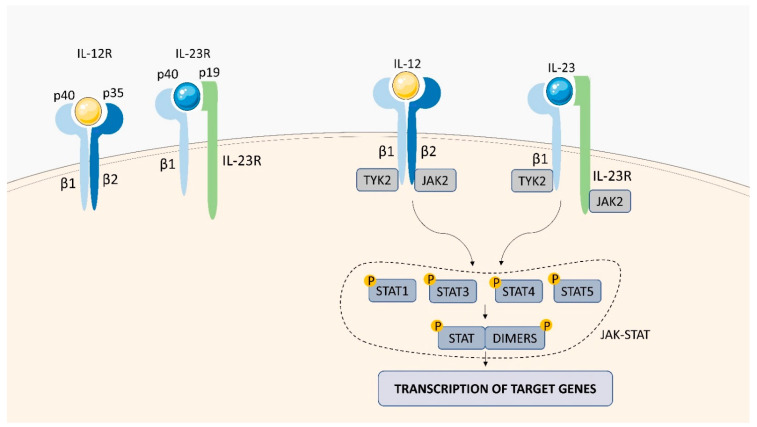
IL-12R and IL-23R structures and signaling. The IL-12 receptor consists of IL-12/23p40 and IL-12p35 subunits, and IL-23 receptor is formed by IL-23p19 and IL-12/23p40 subunits. The interaction of IL-12/23 with IL-12R/23R activates the tyrosine kinases JAK2 /TYK2. The JAK-STAT pathway is activated, which stimulates the transcription of genes in the cell nucleus, leading to the production of cytokines.

IL-12 is a key immunoregulatory cytokine consisting of two covalently-linked subunits, IL-12p35 (35 kDa) and IL-12p40 (40 kDa), each expressed on different chromosomes [27]. The cytokine is involved in the induction of interferon (IFN)-gamma (γ) production and differentiation of CD4^+^ T cells into the type 1 T helper (Th1) phenotype, which is important for protective cell-mediated immune responses against a variety of intracellular pathogens [67]. The biological activities of IL-12 are mediated via binding to the membrane IL-12 receptor (IL-12R) complex, which is composed of two chains: IL-12Rβ1 (CD212) and IL-12Rβ2 (Figure 6) [27]. Binding of IL-12p40 and IL-12p35 subunits to IL-12Rβ1 and IL-12Rβ2 is followed by the activation of JAK kinases (Tyk-2 and Jak-2). Phosphorylated IL-12Rβ2 serves as a docking site for STAT4 proteins, which are phosphorylated by the JAK kinases on their tyrosine residues. To regulate IL-12-related gene transcription, phosphorylated STAT4 proteins are homodimerized and translocated to the nucleus.

IL-12R expression was found to be important for Th1 IFN-γ-producing cells maturation [68]. Zhang et al. found that the percentage of T cells expressing IL-12Rβ1 and IL-12Rβ2 as well as the levels of IL-12Rβ2 mRNA in peripheral blood mononuclear cells stimulated with *Mtb,* were significantly lower in patients with TB compared to controls [69]. On the contrary, the IL-12Rβ2 mRNA expression in patients with active TB was increased in the pleural fluid and lymph nodes. The use of anti-IL-10 and anti-TGF-β antibodies enhanced IL-12Rβ1/IL-12Rβ2 expression and IFN-γ production by *Mtb*-stimulated peripheral blood T cells from patients with TB suggesting that increased TGF-β production could reduce IL-12Rβ1 and IL-12Rβ2 expression in active TB. It also provides evidence that the expression of IL-12Rβ1 and IL-12Rβ2 plays a key role in mediating a protective Th1 response against mycobacteria.

#### 2.1.5. IL-23 Receptor (IL-23R)

IL-23 is a heterodimeric cytokine, that is structurally related to IL-12. Both cytokines share a common IL-12p40 subunit, which, in IL-23, forms a biologically active complex with the IL-23p19 subunit [27,70]. The cytokine induces signals via its specific IL-23 receptor complex formed by two chains: the IL-12Rβ1 subunit, which is also utilized by IL-12, and the unique IL-23R (Figure 6) [70]. Despite the shared subunits, IL-23 and IL-12 have different biological functions. The main function of IL-23 is its ability to stimulate Th17 cells to produce IL-17 and induce proliferation of memory T cells [71]. Like other type I cytokine receptors, the IL-23 receptor complex lacks intrinsic enzymatic activity and is associated with the JAK family members, Janus kinase 2 (Jak2) and tyrosine kinase 2 (Tyk2). Binding of IL-23 to IL-23R promotes JAK kinases activation and phosphorylation of both the kinases themselves and the cytoplasmic tail IL-23R, creating docking sites for STAT3 monomers. Active Jak2/Tyk2 kinases phosphorylate STAT3 monomers, leading to dimerization, nuclear translocation, and DNA binding to target gene promoters [29].

IL-23 can influence both innate and acquired immunity by targeting cells that express IL-23R [72]. TCR γδ T cells are one of the populations highly responsive to IL-23, which, along with IL-17, appears to be a key regulator of immune response in all phases of *Mtb* infection [73]. Shen et al. found that *Mtb* infection in macaques increased the ability of IL-23 to enhance the proliferation of activated Vγ2Vδ2 T cells and production of such cytokines as IL-17, IL-22, IL-2, and IFN-γ [74]. Data from human studies using peripheral blood mononuclear cells (PBMCs) of TB patients showed a dysregulation of the IL-23/IL-17 axis by overexposure to stimulation of *Mtb* antigens [75]. *Mtb*-stimulated CD4^+^ T cells from active TB patients expressed less IL-23R and pSTAT3 than those from latently infected individuals, despite similar levels of IL-23p19 mRNA in *Mtb*-stimulated monocytes. It is therefore suggested that chronic *Mtb* infection disrupts the STAT3 signal transducing pathway in T cells, reducing the signaling effect of IL-23 [76].

### 2.2. Type II Cytokine Receptors

Type II cytokine receptors are transmembrane proteins that bind interferons and members of the IL-10 family. They are structurally related to type I cytokine receptors, but their CHRs have differently arranged conserved cysteine residues and do not contain the WSXWS motif (Figure 2) [77]. Type II cytokine receptors are heterodimers or multimers containing components with high and low ligand affinity. The intracellular domain of the receptors is related to a member of the JAK family, which is activated upon ligand binding [44]. This results in the phosphorylation of the receptors and the formation of docking sites for STAT proteins. Upon phosphorylation by JAKs, STATs form dimers via their SRC homology (SH2) domain and translocate to the nucleus to activate the transcription of target genes. STAT3 and STAT1 factors are activated by all type II cytokine receptors, and some of them also activate STAT2 and STAT5. In addition to activating the JAK/STAT signal transduction system, some receptors also trigger the MAPK signaling pathway [77].

#### 2.2.1. Interferon (IFN)-Gamma (γ) Receptor (IFN-γR)

**Figure 7:**
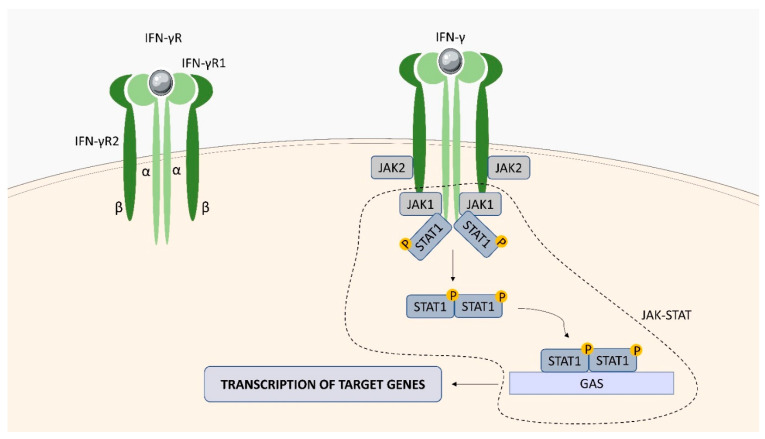
IFN-γR structure and signaling. IFN-γR consists of two β chains (IFN-γR2) and two α chains (IFN-γR1). The interaction of IFN-γ with IFN-γR activates the JAK1/JAK2 tyrosine kinases. The JAK-STAT pathway is activated, which stimulates the transcription of genes in the cell nucleus, leading to the production of cytokines.

IFN-γ, known as type II interferon, exerts a wide range of immunoregulatory activities and is critical for innate and adaptive immunity against infections [78]. The cytokine, secreted primarily by activated T cells and NK cells, acts as a factor promoting macrophage activation, regulating Th1/Th2 balance, and controlling cell proliferation and apoptosis [79]. The heterodimeric IFN-γ receptor (IFN-γR) is composed of two subunits: an α chain (IFN-γR1; CD119), and a β chain (IFN-γR2) (Figure 7). IFN-γR1 binds IFN-γ with high affinity, however it is not capable of mediating biological responses by itself. Activation of the IFN-γR2 subunit is required for the induction of the intracellular signaling pathway [73]. Binding of IFN-γ to IFN-γR1 induces its oligomerization, recruitment of the two IFN-γR2 chains, and activation of the Janus kinases 1 and 2 (Jak1 and Jak2). The activated JAKs phosphorylate a specific C-terminal tyrosine residue in the IFN-γR1 chain that serves as a docking site for STAT1 protein, which undergoes dimerization, migrates to the nucleus and regulates gene expression by binding to gamma-activated sequence (GAS) elements in the promoters of IFN-γ-regulated genes.

Since IFN-γ plays a critical role in the host protective response against mycobacteria, blocking IFN-γR mediated signaling is believed to be an important *Mtb* evasion strategy [80]. IFN-γR gene knockout mice or mice having genetic defects in *IFN-γR* were occurred to be extremely susceptible to *Mtb* infection [81]. In humans, the surface expression of IFN-γR was found to be downregulated in macrophages and peripheral blood mononuclear cells of patients with active TB, which was related to lower IFN-γR mRNA transcription [82]. This was associated with altered expression of the transcription factor Sp1, which is required for *IFN-γR* gene transcription. The resulting phenotype made macrophages resistant to the protective effect of IFN-γ, despite its presence at optimal levels [83].

#### 2.2.2. IL-10 Receptor (IL-10R)

**Figure 8:**
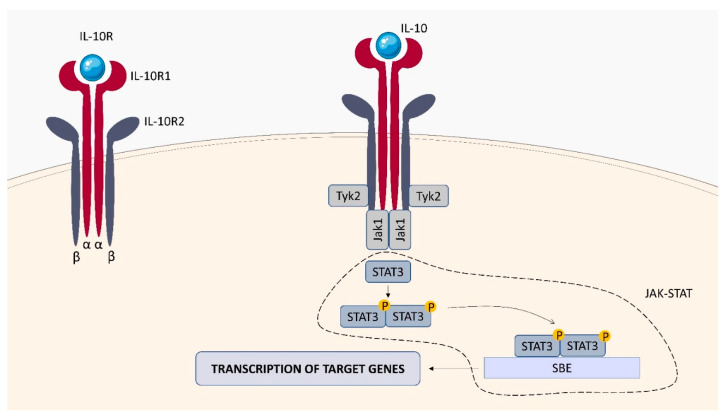
IL-10R structure and signaling. IL-10R consists of two β chains (IL-10R2) and two α chains (IL-10R1). The interaction of IL-10 with IL-10R activates the JAK1/TYK2 tyrosine kinases. The JAK-STAT pathway is activated, which stimulates the transcription of genes in the cell nucleus, leading to the production of cytokines.

IL-10 is a potent cytokine with multiple pleiotropic effects on immunoregulation [84]. It is produced predominantly by leukocytes including T and B lymphocytes, monocytes, macrophages, and DC, as well as by some epithelial cells. The anti-inflammatory activity of IL-10 includes, inter alia (i.a)., downregulating the expression of MHC class II molecules and co-stimulatory molecules on monocytes and macrophages, as well as reducing the production of pro-inflammatory cytokines and chemokines [85]. Dysregulation of IL-10 production is associated with increased immunopathology in response to infection as well as an increased risk of developing various autoimmune disorders [85]. The IL-10 receptor (IL-10R) complex is composed of two subunits, IL-10Rα (IL-10R1), a ligand-binding subunit, and IL-10Rβ (IL-10R2), a signalling subunit (Figure 8). The IL-10Rα chain is specific to IL-10, however the IL-10Rβ subunit is shared by the receptors for other type II cytokines such as IL-22, IL-26, and IFN-lambda (IFN-λ) [86]. Binding of IL-10 to the extracellular domain of IL-10Rα activates phosphorylation of the JAK family kinases, Jak1 and Tyk2 (tyrosine kinase-2). These kinases phosphorylate specific tyrosine residues in the intracellular domain of the IL-10Rα chain, which serve as temporary docking sites for the signal transducer and activator of transcription 3 (STAT3) factor. The Jak1 and Tyk2 phosphorylate STAT3, leading to its homodimerization and subsequent translocation to the nucleus, where it binds to STAT3-binding elements (SBE) in promoters of various IL-10-responsive genes and drives the expression of anti-inflammatory mediators [86,87].

Many studies have shown that IL-10 negatively regulates the immune response during *Mtb* infection [88,89,90,91]. Beamer et al. demonstrated in a mouse model that blockade of IL-10 activity with anti-IL-10R1 antibodies (αIL-10R1) during the first 21 days of *Mtb* infection resulted in increased recruitment of Th1 lymphocytes to the lungs and improved control of bacterial load [90]. This effect was found to be associated with the formation of mature fibrotic granulomas [91]. Furthermore, Pitt et al. revealed that, for 3 weeks following vaccination with *M. bovis* BCG, the IL-10R1 signal blockade enhanced antigen-specific Th1, Th17, and innate lymphoid IFN-γ and IL-17 responses in the lungs that subsequently increased protection against *Mtb* for up to 16 weeks after infection [92]. The results by Dwivedi et al. confirmed that a single dose of αIL-10R1 delivered simultaneously with the BCG vaccine was capable of maintaining long-term control of *Mtb* infection by reducing the pro-inflammatory cytokine profile in the lungs and increasing the production of antigen-specific IFN-γ and IL-17 [93]. These studies indicate the key role of IL-10R1 blockade in the establishment of long-term antigen-specific memory immunity.

#### 2.2.3. IL-22 Receptor (IL-22R)

**Figure 9:**
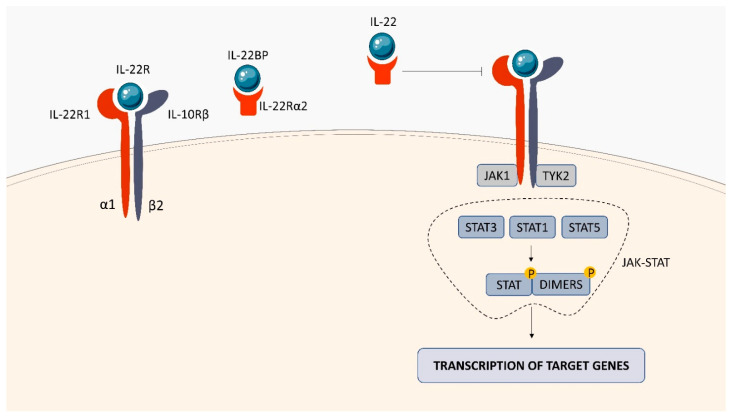
IL-22R structure and signaling. IL-22R consists of 2 subunits (IL-22α1 and IL-10b2). Interaction of IL-22 with IL-22R activates JAK1/TYK2 tyrosine kinases. The JAK-STAT pathway is then activated, which stimulates the transcription of genes in the cell nucleus, leading to the production of cytokines.

IL-22, a member of the IL-10 family, is a potent mediator of cellular inflammatory response [94]. The cytokine is produced by many types of immune cells including activated T cells, innate lymphoid cells (ILCs) and NK T cells [95,96]. IL-22 acts via a heterodimeric receptor complex composed of the IL-22α1 and IL-10β2 subunits [97]. The cytokine binding to the IL-22 receptor complex activates the Janus kinases, Jak1 and Tyk2, followed by phosphorylation and activation of STAT3, STAT1 and STAT5 proteins that migrate to the nucleus, induce expression of specific genes, and trigger the biological activity of IL-22 (Figure 9). IL-22 bioactivity may be negatively regulated by a soluble IL-22Rα2 receptor, known as the IL-22 binding protein (IL-22BP), which prevents binding of the cytokine to the functional cell receptor complex and neutralizes its activity.

Although the IL-22 receptor complex is predominantly expressed on epithelial cells at mucosal sites, recent studies have found that *Mtb*-infected macrophages also express IL-22R [98,99,100,101]. Treerat et al. demonstrated that IL-22R was expressed on macrophages accumulated in tuberculous granulomas in the lungs and that IL-22 could directly induce TNF-α production and macrophage activation to control the infection [102]. Reduced circulating IL-22 levels and a lower percentage of *Mtb*-specific IL-22-producing T cells in TB patients suggest an important role for IL-22 in TB immunology. However, it is still unclear whether the role of IL-22 in antimycobacterial immunity is protective or pathological.

### 2.3. Tumor Necrosis Factor (TNF) Receptor (TNFR) Superfamily

**Figure 10:**
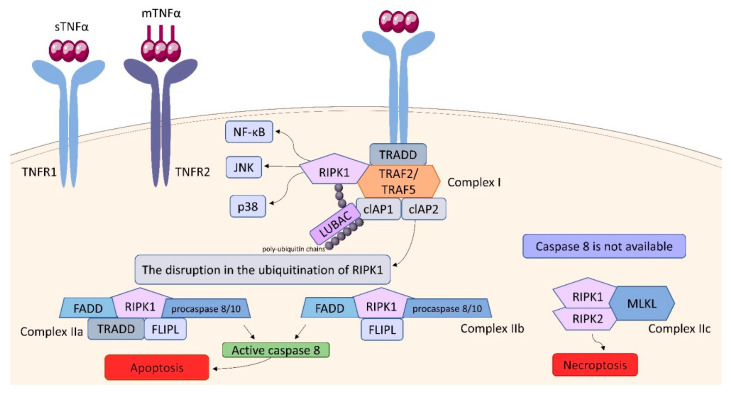
TNFR structure and signaling. Binding of TNF-α to cell surface receptors engages multiple signal transduction pathways, including the IKK/NF-κB, JNK/AP-1 and p38 MAP signaling cascades. TNF also can trigger apoptosis via caspase-8 or necroptosis by activating intracellular RIPK kinases.

Within the TNFSF/TNFRSF superfamily consisting of 19 ligands and 29 receptors, TNF-α and its two main receptors: TNFR1 (TNFRp55/CD120a) and TNFR2 (TNFRp75/CD120b) are among the best characterized members. Soluble TNF-α (sTNF-α) preferentially binds to TNFR1, while membrane bound TNF-α (mTNF-α) is attached to TNFR2 [103,104,105,106,107]. Both receptors are single transmembrane glycoproteins [108]. Similarly to TNF-α, both TNFR1 and TNFR2, are synthesized in a membrane-bound form, which can be cleaved by metalloproteases to release soluble receptors (sTNFR) [109]. TNFR1 is expressed on nearly all nucleated cells [110]. As a death domain (DD) is present in the structure of TNFR1, this receptor belongs to cell sensors termed death receptors (DR) [111]. Two types of the DR signaling complex have been distinguished: the first group comprises the death-inducing signaling complexes (DISCs) and the second one, including TNFR1, transduces both apoptotic and survival signals [112]. TNFR1 is constitutively expressed on most cell types and undergoes activation not only by sTNF-α but also by mTNF-α [111]. TNFR1 stimulation is associated with the formation of two signaling complexes: Complex I, which leads to cytokine signaling and cell survival via activation of NF-kB, JNK, and p38 pathways, Complex II (in different variants) leading to apoptotic or necrotic cell death [111,113] (Figure 10). TNF binding toTNFR1 entails trimerization of the receptor and formation of a core signaling complex within the cytoplasmic tail of TNFR1. Trimerization enables the recruitment of TRADD (TNFR1-associated death domain) through DD. Acting as a scaffold, TRADD recruits the next elements of the forming complex: receptor-interacting serine/threonine-protein kinase 1 (RIPK1) and TNF receptor-associated factor (TRAF) 2, or TRAF5, whereby TRAF2 is responsible for recruiting other elements: cellular inhibitor of apoptosis protein (cIAP) 1 and cIAP2. Thus, the core signaling complex formed on the membrane consists of TRADD, TRAF2 (or TRAF5), RIPK1, and cIAP1/2. There are two factors determining whether it will be a platform for developing Complex I or Complex II: ubiquitination of RIPK1 and the availability of caspase molecules. As a result of the activity of TRAF and cIAP1/2, the ubiquitin chains are added to RIPK1 acting as scaffolds for other signaling factors, including K63, K11, K48 poly-ubiquitin chains and the linear ubiquitin chain assembly complex (LUBAC). The final stage in the formation of Complex I is the attachment of an M1 polyubiquitin chain to RIPK1 by LUBAC. Fully assembled Complex I activates NF-kB, JNK, and p38 pathways via recruitment of the inhibitor of the IkB kinase (IKK) complex and the TGFβ-activated kinase 1 (TAK1)-dependent mechanism, as it was described in detail by Gough and Myles [111]. The disruption in the ubiquitination of RIPK1 resulting in its release into cytosol leads to the formation of Complex II, which can be assembled in two variants: IIa and IIb. The attachment of TRADD, Fas-associated death domain (FADD), FLICE-like inhibitory protein (FLIPL) and procaspase 8 or 10 to RIPK1 forms Complex IIa. Variant IIb includes the same components as Complex IIa, except it lacks TRADD. The assembly of these proteins allow converting procaspase 8/10 to the active form that triggers the cell death by apoptosis. If caspase 8 is not available, Complex IIc is formed. It involves RIPK1 and RIPK3 assembling in an amyloid-like structure to form the necrosome, which leads to cell lysis (necroptosis) via mixed-lineage kinase domain-like protein (MLKL) and phosphoinositides binding [111]. It is suggested, that the effectiveness of complex II formation, caspase-8 activation, and the availability of FLIP in the cell cytosol, which prevents procaspase-8 activation at Complex II, determine whether a cell stays alive/active or dies. This model seems to be an attractive framework for making life-or-death decisions, however it requires more experimental proof [104].

TNFR2 is only present in specific cell subpopulations, including DC, monocytes, and T cells [114,115,116,117,118,119] and after induction in a variety of peripheral B-cell subpopulations [120]. While TNFR1 typically induces cell death and chronic inflammatory mechanisms via canonical NF-κB activation, TNFR2 promotes pro-survival and reparative cascades via the activation of the phosphatidylinositol 3-kinase (PI3K)/serine/threonine protein kinase B (PKB/AKT) pathway and non-canonical NF-κB signaling [105]. TNFR2 lacks DD and after binding TNF-α forms a trimer, which recruits TRAF2 and TRAF1 or TRAF3. Additionally, cIAP1/2, K63 and M1 poly-ubiquitin chains are required for the signaling cascade, which suggests a mechanism of canonical NF-kB activation, resulting in the expression of pro-inflammatory genes [111]. However also the non-canonical activation leading to cell survival and proliferation occurs in a sustained manner. The central component of that pathway is NIK (NF-κB-inducing kinase), which forms an inhibitory complex with TRAF2/3 and cIAP 1/2. The recruitment of TRAF2/3 to TNFR2 causes a disruption of NIK inhibitory complex. Once NIK is activated, the accumulation and activation of IKKα leads to p52 generation via proteolytic degradation of the precursor protein p100, which results in active NF-kB p52/RelB assembling [111]. Although TNFR1 and TNFR2 are characterized by distinct affinity to TNF-α and differential signaling pathways that lead to divergent immune effector functions, the crosstalk between the receptors may occur. The p100 and RelB proteins, overexpressed during the activation of the canonical NF-kB pathway induced by TNFR1, enter the non-canonical activation pathway induced by TNFR2, which results in cell survival and proliferation [111,120]. Numerous studies have shown that TNF plays a primary role in mounting defense mechanisms against *Mtb* infections [121,122,123,124]. Signaling delivered through TNFR2/mTNF-α ensures intercellular communication and it is linked to acute *Mtb* infection controlling, whereas long-term infection control additionally requires soluble TNF [104]. The primary role in the development of the immune response against *Mtb* is attributed to TNFR1, while TNFR2 down-modulates protective immune function, through shedding and neutralization of bioactive TNF [122,123]. Olleros et al. reported that mTNF/TNFR interaction might trigger efficient bactericidal mechanisms. Using transgenic mice model, it was shown, that mTNF was able to activate an efficient immune response against BCG and acute *Mtb* [124]. Due to its ability to induce granuloma formation and IFN-γ expression, mTNF is found to be a key player in the reduction of mycobacterial load and maintaining the cellular activation. An experiment conducted by Segueni et al. with the use of C57BL/6 mice with TNFR1 inactivation in myeloid cells or T lymphocytes revealed that the TNF/TNFR1 pathway plays a prominent role in the activation of innate macrophages and neutrophil myeloid cells [104]. Using transgenic p55ΔNS/p75−/−, p55ΔNS and p75−/− C57BL/6 mouse strains it was found that sTNFR1 remains unemployed during immune regulation associated with the chronic stages of primary *Mtb* infection. However, TNFR1 together with sTNFR2 plays a crucial role in immune regulation of latent TB reactivation [109]. Recent studies show, that anti-*Mtb* chemotherapy may affect the expression of TNF receptors on CD4^+^ lymphocytes [121]. A 6-month therapy, both in drug sensitive (DS)- and drug resistant (DR) TB patients, was found to result in a decreased frequency of conventional T regulatory (cTreg) mTNF+, CD4^+^mTNFR1^+^ and CD4^+^mTNFR2^+^, however the decreased frequency of activated CD4^+^ mTNF^+^ and CD4^+^ mTNFR2^+^ was noticed only in DR-TB patients. Nevertheless, the levels of TNF, IFN-γ and IL-12 remained high and CD4^+^ T cells were characterized by upregulation of TNF and TNFR2. Thus, it is postulated to consider the frequency of activated CD4^+^mTNFR2^+^ cells and inflammatory status in the follow-up of therapy in DR-TB patients [121].

### 2.4. Transforming Growth Factor (TGF)-Beta (β) Receptor (TGFBR) Family

**Figure 11:**
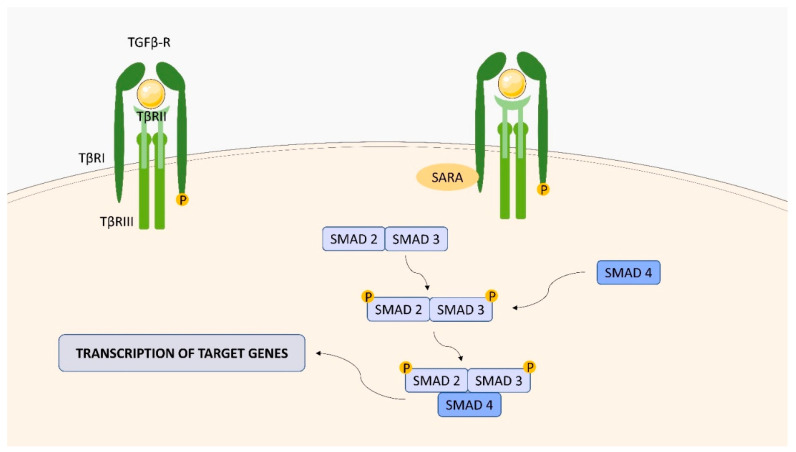
TGFBR structure and signaling. TGFBR consists of three subunits (TBRI, BRII, and BRIII). SMAD2 and SMAD3 are recruited to the type I receptor by the SMAD anchor for receptor activation (SARA).

TGF-β is a multifunctional growth factor regulating cell differentiation, proliferation, migration, apoptosis, and extracellular matrix remodeling [125]. Many cell types, including macrophages, release the cytokine in a latent form that is complexed with two other polypeptides, latency-associated peptide (LAP), and latent TGF-β binding protein (LTBP) [125]. The release of active TGF-β from the complex is catalyzed by serum proteinases such as plasmin. TGF-β receptors are divided into three types: TGF-β type I receptors (TBRI), and TGF-β type II receptors (TBRII), which are serine-threonine kinase transmembrane receptors, and TGF-β type III receptors (TBRIII) [126]. So far, seven TBRIs (known as activin-like (ALK) receptor kinases), ALK1-7, five TBRIIs (TGFBR2, BMPR2, ACVR2, ACVR2B and AMHR2) and two TBRIIIs (betaglycan and endoglin) have been discovered [127,128]. TGF-β signaling is initiated by the binding of the cytokine to TBRII (TGFBR2), which recruits and phosphorylates TBRI (ALK5) (Figure 11). Activated TBRI recruits and phosphorylates receptor-regulated R-SMAD proteins, SMAD2 and SMAD3, which form a complex with a co-mediator SMAD (coSMAD), SMAD4 [129]. SMAD2 and SMAD3 are recruited to the type I receptor by SMAD-anchor for receptor activation (SARA). The R-SMAD/coSMAD complexes are then translocated into the nucleus, where they act as transcription factors and control the regulation of target gene expression. TBRIII, lacking kinase signaling motifs, acts as an accessory receptor modulating the interaction of TGF-β with TBRI/TBRII [130].

TGF-β with its signaling through TBRI/TBRII receptor complex has been linked to the pathogenesis of TB. The cytokine exhibits a wide spectrum of immunomodulatory functions, including down-regulation of production of proinflammatory cytokines such as IL-1, IL-6, and TNF-α, inhibition of T cell and B cell proliferation, attenuation of generation and cytotoxicity of natural killer cells and T cells [131,132]. Bonecini-Almeida et al suggested that the increased expression of TBRI/TBRII and TGF-β observed in patients with active TB, which prevents an excessive T-cell and macrophage response, favors the maintenance of the immunosuppressive environment in granulomas, the primary site of mycobacterial replication [133]. Adams et al found that *Mtb*-infected mice with a T cell-specific conditional deletion of TGF-β receptor showed lower bacterial loads early during infection [134]. Moreover, *Mtb*-specific TGF-βRII cells from the knockout mice produced more IFN-γ during *Mtb* infection, suggesting that targeting the TGF-β receptor could pave the way for new host-directed immunotherapeutics for TB [134].

### 2.5. IL-1 Receptor (IL-1R) Family

**Figure 12:**
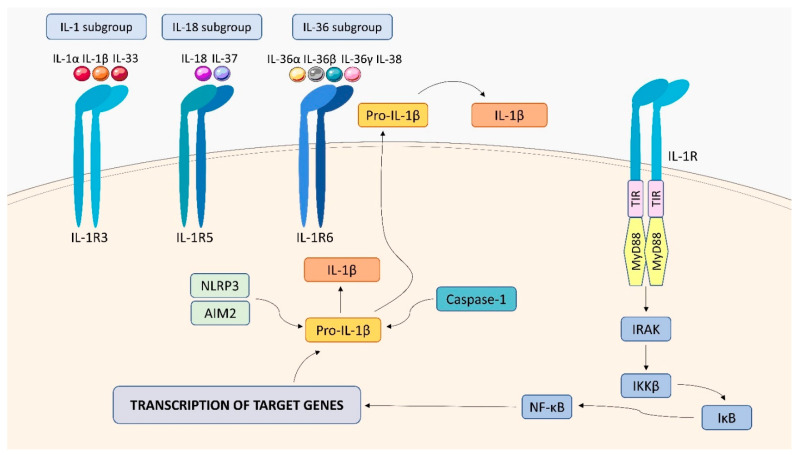
IL-1 family receptors structure and signaling. Receptors of the IL-1 family include IL-1R3, IL-1R5, and IL-1R6. Attachment of the appropriate cytokine to a specific receptor pair triggers a cascade of reactions, resulting in the activation of the transcription factor NF-κB, which stimulates gene transcription in the cell nucleus, leading to the production of cytokines. IL-1 β is produced as an inactive precursor, and its activation requires the action of: NLRP3 and AIM2 and caspase 1.

The IL-1R family consists of 10 type-1 transmembrane proteins with a similar structure, which includes three Ig-like domains (D1, D2, and D3) responsible for ligand binding in an extracellular portion, a transmembrane domain, and an intracellular portion with the Toll-IL-1-receptor (TIR) domain, important for the initiation of signaling [135,136,137]. Based on the functions and differences in the structural features, the members of IL-R family are divided into 4 groups: (1) ligand-binding chains (IL-1R1, IL-1R2, IL-R4, IL-1R5, IL-1R6), (2) accessory chains (IL-1R3, IL-1R7), (3) inhibitors of signaling(IL-1R2, IL-1R8, IL-18BP), and (4) orphan receptors(IL-1R9, IL-1R10) [113] (Figure 12). IL-1R1 binds IL-1α, IL-1β, and IL-1Ra using IL-1R3 as an accessory chain.IL-1R4 is a ligand-binding receptor for IL-33 and forms the IL-33R complex with IL-1R3 as a co-receptor. IL-1R5 binds IL-18 forming the IL-18R complex with the accessory protein IL-1R7, and weakly binds to IL-37 without recruiting IL-1R7. IL-1R6 is a ligand-binding receptor for IL-36 (α, β, and γ) and IL-38 [136,137,138]. Activation of cells by IL-1 family cytokines requires interaction between specific receptors and specified co-receptors. Attachment of the cytokine to a specific pair of receptors results in a cascade of reactions in which the TIR domain is recruited and binds to the myeloid differentiation factor 88 (MyD88), which mediates signal transduction to IL-1 receptor-associated kinases (IRAKs) followed by the phosphorylation of the IκB kinase β (IKKβ). The subsequent steps of the process result in the activation of the NF-κB transcription factor, which stimulates gene transcription in the nucleus leading to the production of cytokines. It should be noted that pro-inflammatory cytokines from the IL-1 family, such as IL-1 β or IL-18, are produced in the form of inactive precursors, and their full activation requires the action of inflammasomes (NLRP3 and AIM2) and caspase 1 or the proteolytic activation of the cytokine in the extracellular environment [136].

The IL-1R pathway is essential for host defense during mycobacterial infection. Fremond et al. revealed that the absence of IL-1R1 led to aa severe defect in the response to acute *Mtb* infection and suggested that IL-1R pathway is an essential component of the myeloid differentiation primary response 88 (MyD88)-mediated signaling, which leads to the development of innate response to *Mtb* [139]. Moreover, IL-1R−/− mice were found to be more susceptible to pulmonary TB, as shown by their higher mortality and an increased mycobacterial burden in the lungs, which was associated with defective granuloma formation, with fewer macrophages and lymphocytes but more granulocytes [140]. Lymphocytes were present mainly in the perivascular areas, suggesting a defective cell migration to inflamed tissue. The impaired host defense in IL-1R−/− mice was also characterized by a decrease in the ability of splenocytes to produce IFN-γ.

### 2.6. Chemokine Receptors

#### 2.6.1. CXCR3

**Figure 13:**
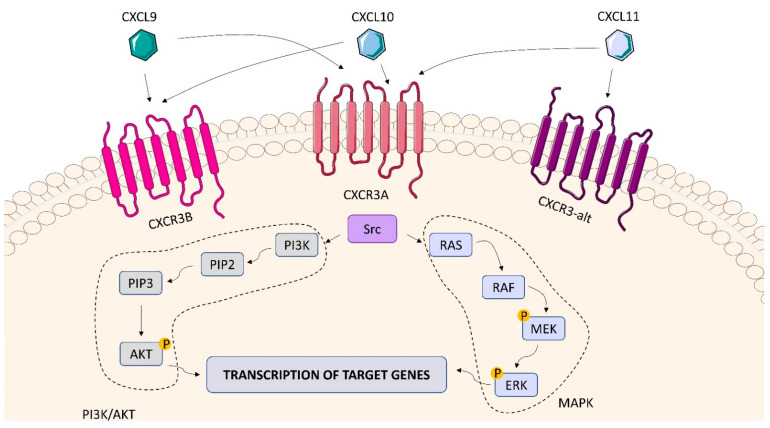
CXCR3 structure and signaling. CXCR3 receptors include CXCRB, CXCXRA, and CXCR-alt. The interaction of chemokines (CXCL9, CXCL10 and CXCL11) with CXCR3 activates the Src, Pi3K and MAPK signaling pathways, which stimulate the transcription of genes in the cell nucleus, leading to the production of cytokines.

Chemokines are a large group of soluble proteins (8–10 kDa) that play an important role in the recruitment and migration of immune cells to inflammatory sites. CXCL10, formerly called IP-10 (interferon gamma induced 10kDa protein), is a ligand of the chemokine receptor CXCR3 which plays a role in various physiological and pathological processes regulated by Th1 cells [141]. CXCR3 binds α-chemokines CXCL9, CXCL10 and CXCL11. It is primarily expressed on activated T cells (mainly CD4^+^ Th1 cells), B cells, NK cells and DC. Due to the difference at the amino end of the receptor, CXCR3 can be divided into three different splice variants: CXCR3A, CXCR3B and CXCR3-alt (Figure 13). CXCR3 is linked to several signaling pathways including Src, PI3K and MAPK [142]. *Mtb* antigens induce inflammatory response contributing to IFN-γ production. IFN-γ-induced IP-10 binds to the CXCR3 receptor triggering chemotactic activity and directing leukocytes to the inflammatory site where *Mtb* can be ultimately killed [143]. CXCR3 is a seven-transmembrane G-protein-coupled receptor. The DRY motif is crucial for the engagement of cytoplasmic G proteins to initiate signaling. CXCR3 binds three different ligands, namely CXCL9, CXCL10 and CXCL11. These ligands bind to specific and distinct regions within the receptor [144]. In *Mtb*-infected macaques, CXCR3+ Th1 lymphocytes have been shown to efficiently settle the lung parenchyma during active TB [145]. Using immunofluorescence staining and quantification of CXCR3^+^ CD4^+^ cells in lung tissue sections from animals with asymptomatic LTBI and active TB, it was confirmed that CXCR3^+^CD4^+^ cells were mainly localized in granulomatous areas. The number of CXCR3^+^CD4^+^ cells in granulomas inversely correlated with *Mtb* burden, suggesting that these cells may have protective functions [146]. Studies on C57BL/6 mice and BALB/c mice with the reduced expression of the CXCR3 receptor showed that 30 days post aerosol infection with *Mtb* there were marked differences in bacterial burden in the lungs and spleen in favour of CXC3−/− mice compared to the wild-type mice [147,148]. It may suggest that the absence of CXCR3 enables the host to control chronic TB infection with an established bacterial burden. However, although the bacterial load in CXC3−/− mice was lower compared to that in the control animals, they were not able to clear the infection completely [148]. CXCR3 deficiency per se did not alter the overall distribution of leukocytes in the lungs of *Mtb*-infected mice, but an alteration in their diversity was observed - particularly in terms of CD4^+^/CD8^+^ lymphocyte and neutrophil ratios. The CD4^+^ /CD8^+^ T-lymphocyte ratio of CXCR3−/− BALB/c mice was 2.3, whereas that of wild-type mice was 1.1. After 6 months, the ratios were 3.6 and 1.0, respectively. The number of neutrophils in the lungs of CXCR3−/− BALB/c mice infected with *Mtb* was lower than that of wild-type mice at all time points analyzed [148]. The interaction between CXCR3 and IP-10 may adversely affect T cell stimulation by APCs via disrupting the immune synapse formation [149]. Shang et al. showed that the levels of CXCR3, IFN-γ and IP-10 in the blood of patients with spinal TB were significantly higher compared to those found in healthy subjects [150]. The results of Yu et al. indicated that the expression of CXCR3 or CCR4 on CD4^+^ T cells might serve as a potential diagnostic marker in TB [151]. Similarly, Lee et al. showed that CXCR3 ligands could be useful surrogate markers for the diagnosis of active TB and clinical evaluation of TB patients [152].

#### 2.6.2. CXCR1 and CXCR2

**Figure 14:**
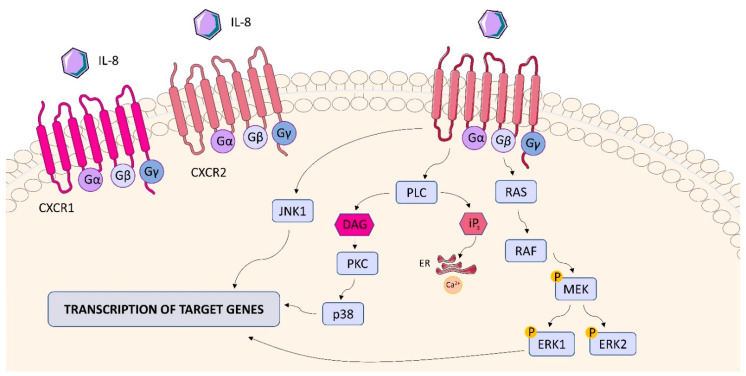
CXCR1 and CXCR2 structure and signaling. The interaction of IL-8 with CXR1/CXCR2 activates ERK1/2, JNK1, and p38 and induces rapid intracellular Ca^2+^ mobilization. ERK1/2, JNK1, and p38 stimulates transcription of genes in the cell nucleus, leading to the production of cytokines.

Interleukin-8 (IL-8, CXCL8) is produced in the lung in response to *Mtb* infection. This chemokine, produced by monocytes/macrophages, alveolar epithelial cells and even fibroblasts, is one of the most studied chemokines in the context of TB [3]. The cytokine attracts immune cells to the lung and plays an important role in granuloma formation by acting as a key regulator of host immunity against infection [153]. This inducible pro-inflammatory cytokine belonging to the chemokine family mainly binds to CXCR1 and CXCR2 receptors but also to atypical non-signaling chemokine receptors such as DARC/ACKR1 (Duffy antigen receptor for chemokines/Atypical chemokine receptor 1) [154]. CXCR1 and CXCR2, known as interleukin-8 receptor A (IL-8RA) and interleukin-8 receptor B (IL-8RB), respectively, belong to the GPCR (G Protein-Coupled Receptors) family, whose structure consists of 7 transmembrane domains (Figure 14) [155]. IL-8RA, IL-8RB and IL-8RBP (IL-8RB pseudogene) form a gene cluster in a region located on chromosome 2q33-q36 [156]. CXCL8 binding to CXCR1/2 activates several signaling cascades mediated by G protein. Receptor activation immediately leads to the dissociation of the Gαi subunit from the βγ subunits and subsequently activates growth and stress kinases such as ERK1/2, JNK1, and p38. G protein activation also induces rapid intracellular mobilization of Ca2+ released from the endoplasmic reticulum (ER) and inhibition of adenylyl cyclase, resulting in reduced production of cyclic AMP. CXCR1/2 activation also leads to phosphorylation of the receptor at the C-terminus by GRK2/6 and recruitment of β-arrestin1/2 to mediate receptor internalization. Internalized receptors are either recycled back to the cell surface or directed to lysosomes for degradation [157].

CXCR1 and CXCR2, both present on neutrophils, play an important role in granulocyte recruitment to the site of infection [158]. It is extremely important during the inflammatory response in TB. CXCL8-CXCR1/2 pathways are responsible not only for the recruitment of neutrophils but also for induction of granulocyte effector mechanisms, such as oxidative burst, granule release to eliminate the inflammatory stimulus, as well as intracellular Ca2+ ion mobilization. Thus, these pathways protect the host from further progression of *Mtb* infection. A disruption in the CXCL8-CXCR1/2 pathways can severely affect the host’s immune mechanisms against infection and even lead to death [158,159]. The oxidative burst in neutrophils induced via CXCR1/2 was significantly attenuated in patients with pulmonary TB compared to those with latent TB and healthy individuals. Alaridah et al. demonstrated that TB patients showed a significant increase in CXCR1 expression compared to the controls [160]. In a study by Juffermans et al. peripheral blood granulocytes from TB patients showed reduced expression of CXCR2, but not CXCR1, compared to controls [161]. Meddows-Taylor et al. described reduced expression of both CXCR1 and CXCR2 on granulocytes from pulmonary TB patients with or without HIV infection [162]. Chemokine receptor expression in patients with active and latent TB may therefore potentially have implications for future therapies [160]. CXCR2 is important in the pathology of a wide range of chronic lung diseases, and modulation of CXCR2 function is considered a possible therapeutic strategy. Interestingly, blocking these GPCRs prior to mycobacterial infection abrogated mycobacteria-induced actin redistribution and suppression of epithelial NF-κB and c-Jun, further supporting the theory that mycobacteria use GPCR to manipulate cell signaling [163]. Blocking CXCR2 reduces mycobacteria-induced IL-10 secretion. This cytokine modulates anti-inflammatory mechanisms through targeting NF-κB and may contribute to the suppression of transcriptional activity. Impaired cell activation and recruitment are related to increased IL-10 production and decreased CXCR2 expression under septic conditions [163]. CXCR1 and CXCR2 were also found on monocytes isolated from TB patients and patients with latent TB, implying that CXCR1/CXCR2 expression on immune cells other than neutrophils may play a role during *Mtb* infection [164]. The multitude of processes involving CXCL8 receptors in the course of TB infection indicates their significant role in the *Mtb*-host interplay.

### 2.7. IL-17 Receptor Family (IL-17R)

**Figure 15:**
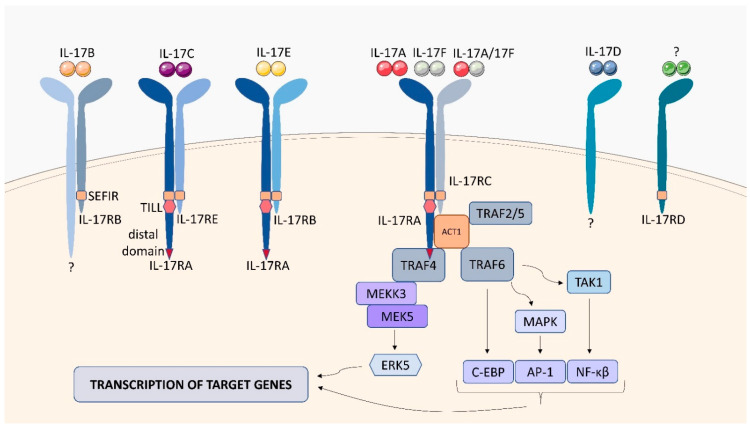
Structure and signaling of receptors of the IL-17 family. IL-17 receptor family includes IL-17RA, IL-17RB, IL-17RC, and IL-17RD, which can exist as homodimers (IL-17RD) and heterodimers (IL-17RB/?,IL-17RA/RE, IL-17RA/RB, IL-17RA/RC). The interaction of IL-17 with IL-17R leads to the activation of NF-κB, MAPK, AP-1, C/EBP, and ERK5, which stimulate the transcription of genes in the cell nucleus, leading to the production of cytokines.

The IL-17 family of cytokines consists of 6 members: IL-17A, IL-17B, IL-17C, IL-17D, IL-E, IL-17F, which play important roles in regulating the innate and adaptive immune response. IL-17 is produced by T helper type 17 (Th17) cells, innate immune cells, and epithelial cells [165]. Receptors of the IL-17 family of cytokines include IL-17RA, IL-17RB, IL-17RC, IL-17RD, which can form heterodimers or homodimers (Figure 15). Cytokines of the IL-17 family transmit signals through IL-17 receptors, which differ from other known cytokine receptors. Most IL-17 family cytokines signal via a heterodimeric receptor comprised of IL-17RA and a second chain which depends on the ligand. The IL-17RA/C receptor complex recognizes the homodimer IL-17A/IL-17A and IL-17F/IL-17F or the heterodimer IL-17A/IL-17F. It is worth noting that IL-17RA binds to cytokines with different affinities, IL-17-RA shows high affinity for IL-17A, 100 times weaker for IL-17F, intermediate for IL-17A/F and weaker for IL-17B, IL-17C, IL-17D, IL-17E. Furthermore, IL-17RC has a higher affinity for IL-17F than for IL-17A. IL-17C signals through heterodimeric receptors IL-17RA/RE, and IL-17E interacts with IL-17RA/RB. IL-17RB recognizes IL-17B and the receptor for IL-17D is still unknown [166]. Each of the IL-17 receptors is a single transmembrane protein that contains conserved structural motifs: the extracellular fibronectin III-like domain as well as a similar expression of the fibroblast growth factor and the IL-17R (SEFIR) domain [167]. Compared to other IL-17 receptors, IL-17RA has two additional domains, a TILL (TIR (Toll/IL-1R) - like loop) domain and a distal domain in the C-terminus. Activation of signaling pathways occurs when IL-17 binds to IL-17R, resulting in the attachment of the adaptor protein Act1, which has E3 ubiquitin ligase activity. Recruitment of TRAF6 and TAK1, leads to the activation of NF-κB, as well as MAPKs and transcription factors, AP-1 and C/EBP (the canonical pathway). Through TRAF-4, Act1 binds to MEKK3 and MEK5 activating ERK5. Both pathways lead to the transcription of inflammatory genes. The stability of IL-17 target genes mRNA is controlled by the binding of TRAF2/5 to Act1.

IL-17A is known to play an important role during *Mtb* infection, but the function of the receptor with which it interacts is not fully understood. Using IL-17RA−/− mice, Frenches et al. demonstrated that the IL-17RA pathway is essential for normal signal transduction during TB infection. An increased susceptibility to *Mtb* infection has been observed in IL-17RA−/− mice, associated with delayed neutrophil recruitment in the early stage of TB due to an abnormal cytokine response (reduced levels of IL-6 and IL-10 and increased IL-1β, in the absence of IL-17RA) [168]. Lombard et al. also demonstrated the effect of the IL-17RA pathway on neutrophil recruitment to the lung during mycobacterial infections. Reduced production of ligands involved in neutrophil recruitment (CXCL-1 for *Mtb* and CXCL1 and CLXCL-5 for BCG) was observed in IL-17RA−/− mice infected with *Mtb* or BCG, which contributed to reduced neutrophil numbers in the lungs of IL-17RA mice [169].

## 3. Implications of Cytokine Receptor Gene Defects for the Course of Mycobacterial Infection

Studies on immunodeficiencies associated with increased susceptibility to mycobacteria have identified several abnormalities in genes encoding cytokine receptors or key molecules in the cytokine signal transduction pathways. These alterations include genes that encode the components of IL-12R (IL-12Rβ1), IFN-γR (IFN-γR1, IFN-γR2), IL-2R (IL-2α), the IFN-γ signal transducer and activator of transcription factor 1 (STAT1) as well as molecules involved in the nuclear factor NF-κB signaling pathway (IRAK4, NEMO).

Defects in the secretion or signaling of type 1 cytokines have been discovered to predispose to infections caused by weakly pathogenic mycobacteria, such as nontuberculous environmental mycobacteria (*M. avium*, *M. abcessus*, *M. fortuitum*, *M. chelonei*, *M. kansasii*, and even *M. smegmatis*) or *Mycobacterium bovis* Bacillus Calmette-Guérin (BCG). It was first demonstrated by Kamijo et al. in IFN-γR-deficient mice, which showed a marked decrease in the production of TNF-α, IL-1α, and IL-6 and a defect in the formation of characteristic granulomas after BCG infection [170]. The first demonstration that genetic defects may contribute to severe mycobacterial infections in humans involved the identification of molecular defects in the IL-12/IFN-γ dependent signalling pathway in patients with Mendelian susceptibility to mycobacterial diseases (MSMD). MSMD is a group of complete or partial primary immunodeficiencies characterized by impaired IFN-γ-mediated immunity [171,172]. In MSMD-affected individuals, weakly virulent mycobacteria are responsible for severe infections [173]. Interestingly, infections with other intracellular bacteria such as *Salmonella* Enteritidis, *Salmonella* Typhimurium or *Listeria monocytogenes*, have been documented [174]. Mutations involved in the MSMD have been found in 7 autosomal genes, which encoded either a chain of the IFN-γ receptor (IFN-γR1 or IFN-γR2), STAT1, the p40 subunit of IL-12/IL-23 (IL-12p40), the ligand-binding chain of the IL-12/IL-23 receptor (IL-12Rβ1), ubiquitin-like protein ISG15 (ISG15), and interferon regulatory factor 8 (IRF8) [173,175]. Moreover, two mutations in X-linked genes, encoding the inhibitor of nuclear factor NF-κβ kinase regulatory subunit gamma (NEMO), and the cytochrome B-245 beta chain (CYBB) have been described [173]. Complete deficiency of either of the two IFN-γR chains or STAT-1 is associated with the development of severe, often fatal, form of the MSMD syndrome in early childhood. On the contrary, partial recessive and dominant IFN-γR1 deficiency and partial recessive IFN-γR2 deficiency as well as complete IL-12p40 and IL-12Rβ1 deficiencies are associated with a milder form and a good clinical outcome [174,175,176,177,178].

## 4. Conclusions

Cytokine receptors play a central role in coordinating immune and inflammatory responses during mycobacterial infection. The key evidence derives from both studies in experimental models and observations in patients with genetic deficiencies in cytokine receptors or their signaling pathways. Understanding their remarkable features provides major insights into the mechanisms of antimycobacterial immunity and can lead to new approaches for immunological intervention in TB and other mycobacterial diseases.

## Figures and Tables

**Table 1:** Cytokine receptor families involved in the antimycobacterial immune response.

Family	Cytokine Receptors	Features	Mechanism of Action	Targeting Genes/Responsive Genes
type I cytokine receptors	IL-2R, IL-4R, IL-6R, IL-12R, IL-23R, G-CSFR, GM-CSFR	- recognize four-helix bundle cytokines - lack intrinsic protein tyrosine kinase activity - contain a WSXWS motif in the extracellular domain - consist of different chains, some of which are involved in cytokine interaction and others in signal transduction	transduction of the signaling pathway through the involvement of the non-receptor Janus kinases (JAKs) and the signal transducer and activator of transcription (STATs) factors	IL-2R: *IL-2*, *CTLA-4*, *Bcl-2*, *cyclins*, *FoxP3*, *FasL*, *CD25* [19] IL-4R: *CD23*, *MHC class II*, *IL-4α*, *GATA3*, *BDNF/NGF*, *YM-1* [20,21] IL-6R: *CIS*, *SOCS*, *Mcl-1*, *TIMP-1*, *Pim-1*, *c-Myc*, *cytokines*, *TFs* [22,23,24,25,26] IL-12R: *IFN-γ*, *TNF-α*, *T-bet*, *PD-1* [27,28] IL-23R: *RIG-I*, *IL-23*, *IL-23R*, *IL-17*, *GM-CSF* [28,29] G-CSFR : *MMP2*, *VEGF*, *GATA-3*, *ATG2A*, and *PIAS3* [30,31] GM-CSFR: *c-Myc*, *IL-1β*, *IL-12p70*, *IL-6*, *TNF-α*, *iNOS* [32]
type II cytokine receptors	IL-10R, IL-22R, IFN-γR	- consist of shared chains and cytokine specific chains - lack a WSXWS motif in the extracellular domain - intracellular domain is typically associated with a tyrosine kinase belonging to the Janus kinases (JAKs)	transduction of the signaling pathway through the involvement of the non-receptor Janus kinases (JAKs) and the signal transducer and activator of transcription (STATs) factors	IL-10R: IL-4, IL-13, IL-1β, IL-6, TNF-α, CD80,CD83, CD86, Bcl-2, Bcl-xL, caspase-3 [33,34] IL-22R: *Bcl-2*, *Bcl-xL*, *MCL-1*, *Cyclin D1*, *CDK4*, *CCL2*, *RANKL2*, *CXCL5*, *S100A7*, *S100A8*, *S100A9*, *lipocalin 2*, *MMP1*, *MMP3* [35] IFN-γR : S100A family, IL-7, TAP1, SERPING1, VAMP5, TNFAIP2, TNFSF10, PARP-1, CD274, CCL2, NOX1, NOX4, IRF2 [36,37]
tumor necrosis factor (TNF) receptor family	TNFR	- soluble TNF-α (sTNF-α) preferentially binds to TNFR1, whereas membrane-bound TNF-α (mTNF-α) to TNFR2 - TNFR1 contains a death domain (DD), while TNFR2 lacks the DD - both receptor types trigger distinct and common signaling pathways	transduction of the signaling pathways through IKK/NF-κB, JNK/AP-1 and p38 MAP signaling cascades. TNF also can trigger apoptosis via caspase-8 or necroptosis by activating intracellular receptor-interacting serine/threonine-protein (RIPK) kinases	*MKP-1*, *MCP-1*, *COX-2*, *IL-6*, *SOD-2* [38]
transforming growth factor (TGF)-β receptor family	TGFBR	- single pass serine/threonine kinase receptors - grouped into three types, TBRI-III (seven TBRI, five TBRII, and two TBRIII receptors)	ligand-bound type II receptors activate type I receptors by phosphorylation, which then autophosphorylate and bind intracellular Sma- and Mad-related proteins (SMAD)	*PDGF-B*, *FoxP3*, *Bcl-xL*, *Beclin 1* [39,40]
chemokine receptors	IL-8R CXCR3	- consist of seven transmembrane domains coupled to a G protein heterotrimer - are divided into families corresponding to the 4 distinct subfamilies of chemokines (CXC, CC, CX3C, and XC)	transduction of the signal by G-protein coupled receptors, which dissociate to activate diverse downstream pathways resulting in cellular polarization and actin reorganization	ICAM-1, VCAM-1, Cox-2 [41]
immunoglobulin (Ig) receptor superfamily	IL-1R family	- three Ig-like domains (D1, D2, and D3) in an extracellular portion, a transmembrane domain, and an intracellular portion with the Toll-IL-1-receptor (TIR) domain	signal transduction through the toll/interleukin-1 receptor (TIR) domain, which recruits MyD88 adaptor protein activating the NF-κB pathway.	*IL-6*, *CCL2*, *IL-8* [39]
interleukin (IL)-17 receptor family	IL-17RA-E	- contain a SEF/IL-17R (SEFIR) subdomain - longer cytoplasmic tail of IL-17RA contains some additional structural domains, such as the TILL domain (“TIR-like loop”) or the inhibitory CBAD domain (“C/EBPβ-activation domain”)	signaling cascade activates the extracellular signal-regulated protein kinase (ERK), the c-jun N-terminal kinase (JNK), and the p38/MAPK pathway	*IL-6*, *IL-1β*, *CXCL1*, *CXCL2*, *G-CSF* [42]

Abbreviations: ATG2A, autophagy related 2A; Bcl-2, B-cell lymphoma 2; Bcl-xL, B-cell lymphoma extra large; BDNF/NGF, brain-derived neurotrophic factor/nerve growth factor; CCL2, C-C Motif Chemokine Ligand 2; CDK4, Cyclin-dependent kinase 4; CIS, cytokine-inducible SH2 containing protein; c-Myc, cellular Myc; COX-2, cyclooxygenase 2; CXCL1, C-X-C Motif Chemokine Ligand 1; CXCL2, C-X-C Motif Chemokine Ligand 2 CXCL5, C-X-C Motif Chemokine Ligand 5; CTLA-4, cytotoxic T-lymphocyte-associated protein 4; FasL, Fas ligand; FCGR, Fc-gamma receptor; FoxP3, forkhead box P3; GATA3, GATA binding protein 3; G-CSF, granulocyte colony-stimulating factor; GM-CSF- granulocyte- macrophage colony-stimulating factor; ICAM-1, Intercellular Adhesion Molecule 1; IL, interleukin; iNOS, Inducible nitric oxide synthase; IRF2, Interferon regulatory factor 2; Mcl-1, Myeloid cell leukemia-1; MCP-1, Monocyte chemotactic protein-1; MHC, major histocompaibility complex; MKP-1, MAP kinase phosphatase 1; MMP1, , matrix metalloproteinase-1; MMP-2, matrix metalloproteinase-2; MMP3, matrix metalloproteinase-3; NOX1, NADPH oxidase 1; NOX4, NADPH oxidase 4; PARP-1, Poly (ADP-ribose) polymerase 1; PD-1, Programmed cell death protein 1; PIAS3, Protein Inhibitor of Activated STAT3; Pim-1, Pim-1 proto-oncogene; PDGF-B, platelet-derived growth factor subunit B; RANKL2, Receptor activator of nuclear factor kappa-Β ligand; RIG-I, retinoic acid-inducible gene I; SERPING1, serpin family G member 1; SOCS, CIS/suppressors of cytokine signaling; SOD-2, superoxide dismutase 2; S100A7, S100 calcium-binding protein A7; S100A8, S100 calcium-binding protein A8; S100A9, S100 Calcium Binding Protein A9; TAP1, Transporter associated with antigen processing 1; T-bet, T-box protein expressed in T cells; TFs, transcription factors; TIMP-1, TIMP metallopeptidase inhibitor 1; TNFAIP2, tumor necrosis factor alpha-induced protein 2; TNFSF10, tumor necrosis factor (ligand) superfamily, member 10; VAMP5, Vesicle-Associated Membrane Protein 5; VCAM-1, Vascular cell adhesion molecule 1; VEGF, Vascular endothelial growth factor.

## Data Availability

The data presented in this study are openly available under reference numbers.
